# Alterations of microbiota structure in the larynx relevant to laryngeal carcinoma

**DOI:** 10.1038/s41598-017-05576-7

**Published:** 2017-07-14

**Authors:** Hongli Gong, Yi Shi, Xiyan Xiao, Pengyu Cao, Chunping Wu, Lei Tao, Dongsheng Hou, Yuezhu Wang, Liang Zhou

**Affiliations:** 1 Shanghai Key Clinical Disciplines of Otorhinolaryngology, Department of Otorhinolaryngology, Eye, Ear, Nose, and Throat Hospital of Fudan University, 83 Fenyang Road, Shanghai, 200031 China; 2 Department of Clinical Laboratory, Shanghai Pudong Hospital, Fudan University Pudong Medical Center, 2800 Gongwei Road, Shanghai, 201399 China; 3 Shanghai Key Laboratory for Reproductive Medicine, Department of Histology and Embryology, Shanghai Jiao Tong University School of Medicine, 280 South Chongqing Road, Shanghai, 200025 China; 4 Shanghai-MOST Key Laboratory of Health and Disease Genomics, Chinese National Human Genome Sequencing Centre, 250 Bibo Road, Shanghai, 201203 China

## Abstract

The microbial communities that inhabit the laryngeal mucosa build stable microenvironments and have the potential to influence the health of the human throat. However, the associations between the microbiota structure and laryngeal carcinoma remain uncertain. Here, we explored this question by comparing the laryngeal microbiota structure in laryngeal cancer patients with that in control subjects with vocal cord polyps through high-throughput pyrosequencing. Overall, the genera *Streptococcus*, *Fusobacterium*, and *Prevotella* were prevalent bacterial populations in the laryngeal niche. Tumor tissue samples and normal tissues adjacent to the tumor sites (NATs) were collected from 31 laryngeal cancer patients, and the bacterial communities in laryngeal cancer patients were compared with control samples from 32 subjects. A comparison of the laryngeal communities in the tumor tissues and the NATs showed higher α-diversity in cancer patients than in control subjects, and the relative abundances of seven bacterial genera differed among the three groups of samples. Furthermore, the relative abundances of ten bacterial genera in laryngeal cancer patients differed substantially from those in control subjects. These findings indicate that the laryngeal microbiota profiles are altered in laryngeal cancer patients, suggesting that a disturbance of the microbiota structure might be relevant to laryngeal cancer.

## Introduction

The human microbiota can be considered an organ composed of mixed species with functions that enable the construction of a polymicrobial assemblage^1, 2^. Microbial communities are abundant and relatively stable in the human body, which is constantly exposed to these microbial factors, and these communities play a fundamental role in regulating the health and physiology of the host via cooperative and competitive interactions^3–5^. Indeed, aberrations in the microbiota profiles play causative roles in the development of many clinical diseases, such as periodontitis, obesity, inflammatory bowel disease, diabetes mellitus, metabolic syndrome, atherosclerosis, and liver cirrhosis^6–12^. Specifically, differences in the relative abundance of certain microbial communities have been observed in cancer patients, indicating that disturbances in this multispecies synergy might be an important factor related to tumorigenesis^13–15^. Recently, the use of animal models possessing the same molecular pathway mechanisms observed *in vivo* has suggested that disruption of the microbiota can promote tumor initiation and development^16–18^.

However, the mechanisms through which microbial factors influence susceptibility to laryngeal carcinoma remain elusive. Laryngeal carcinoma is one of the most common malignancies of the head and neck, and squamous cell carcinoma is the most frequent histological type of laryngeal carcinoma, accounting for 98% of cases^19, 20^. The main risk factors for this cancer include tobacco smoking and alcohol consumption, and the roles of these risk factors have been consistently confirmed by a growing number of studies^21, 22^. The larynx is a part of the upper airway that harbors large populations of *Firmicutes, Proteobacteria*, and *Bacteroidetes*; however, the profiles of the bacterial communities at this site are different from those at other anatomical locations in the respiratory system^23^. A recent report indicates that *Porphyromonas gingivalis* can induce expression of the B7-H1 and B7-DC receptors in oral squamous cell carcinoma (OSCC), which can reduce T cell proliferation and facilitate immune evasion^24^. The altered expression of B7-H1 might induce the onset of chronic inflammatory disease, which frequently precedes the development of human cancers^24^. This bacterium might induce proenzyme matrix metalloproteinase 9 (proMMP9) expression by activating the ERK1/2-Ets1, p38/HSP27, and PAR2/NF-kB pathways^25^, and the overexpression of proMMP9 is able to promote the cellular invasion ability of OSCC cells^25^. The nature of microbial synergy in the human larynx niche is not thoroughly understood to date. In our previous pilot study, we revealed the structure of the microbial communities in the larynx by examining 16S rRNA gene variable regions 1 and 2 (V1 and V2), but our interesting findings require further confirmation^26, 27^. In addition to studies focused on increasing our understanding of the structure of the bacterial communities in the larynx, an investigation of the associations between the microbiota patterns and laryngeal carcinoma is critical.

This study aimed to gain a better understanding of the ecology of the laryngeal communities in laryngeal carcinoma patients and control subjects and to elucidate the relationships between the microbiota characteristics and laryngeal carcinoma. Specifically, we performed high-throughput pyrosequencing of the 16S rRNA gene to identify the laryngeal microbiota in patients with laryngeal carcinoma and vocal cord polyps. By analyzing the 16S rRNA gene variable region 3 (V3), we compared the microbiota structures in the tumor tissues and the normal tissues adjacent to the tumor sites (NATs) from laryngeal squamous cell carcinoma (LSCC) patients and in control tissues from subjects with vocal cord polyps.

## Results

### Clinical samples and sequencing data quality

Tumor tissues were collected from 31 LSCC patients. Due to the limited negative margin between the tumors and normal sites in the larynx, we acquired only 24 NATs from the LSCC patients. Control tissue samples were obtained from 32 subjects with vocal cord polyps. The clinical characteristics of the subjects enrolled in the study were provided (see Supplementary Table S1). After processing the dataset of bacterial V3 sequences from the 16S ribosomal RNA gene, we obtained 176,757 sequences, with an average of 2,032 ± 1,466 (ranging from 617 to 6,770) sequences per sample and an average sequence length of 141 ± 7 bp. We obtained 8,887 operational taxonomic units (OTUs) after assigning the sequences to species-level OTUs using a 97% pairwise-identity cut-off. The high-quality dataset was verified by evaluating the Chao1, ACE, Simpson, Shannon, Evenness, and Good’s coverage indices (see Supplementary Table S2).

### Bacterial communities in the larynx niche

**Figure 1:**
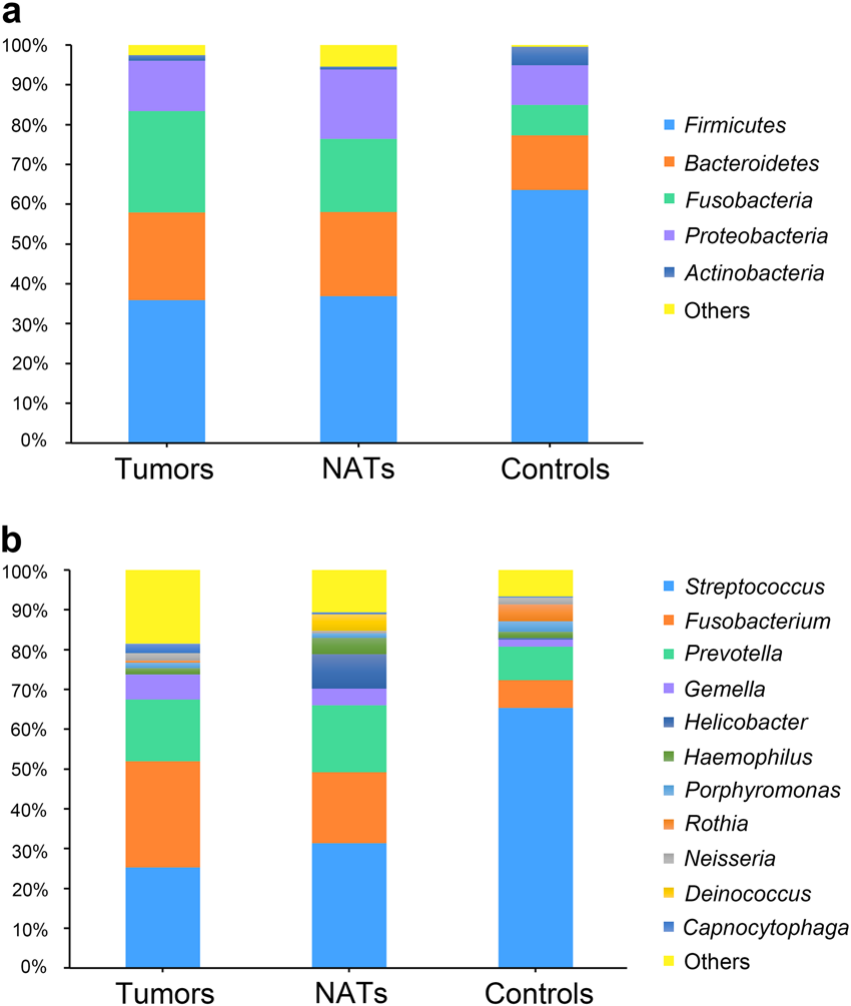
Abundances of dominant bacterial communities in the larynx. (**a**) Abundances (% of total 16S rRNA sequences) of the predominant bacterial phyla in the tumor tissue samples and the normal tissues adjacent to tumor sites (NATs) from laryngeal carcinoma patients, and in control tissue samples from vocal cord polyps subjects. (**b**) The Main bacterial genera in the laryngeal mucosa of the three groups of samples.

The overall community patterns revealed a broad range of taxa with different abundances detected in the larynx. The phyla *Firmicutes* (46.4%), *Bacteroidetes* (18.7%), *Fusobacteria* (16.9%), *Proteobacteria* (13.0%), and *Actinobacteria* (2.4%) accounted for the majority of microbial communities. At the genus level, *Streptococcus* (41.7%), *Fusobacterium* (17.0%), *Prevotella* (13.2%), *Gemella* (4.1%), *Helicobacter* (2.6%), and *Haemophilus* (2.3%) were the dominant populations (see Supplementary Fig. S1). The prevalent bacterial phyla and genera of three groups of laryngeal samples (Fig. 1), and the relative abundances of the 25 most common genera from 87 tissue samples were displayed (see Supplementary Fig. S2).

### Comparison of the microbiota structures of LSCC patients and control subjects

**Figure 2:**
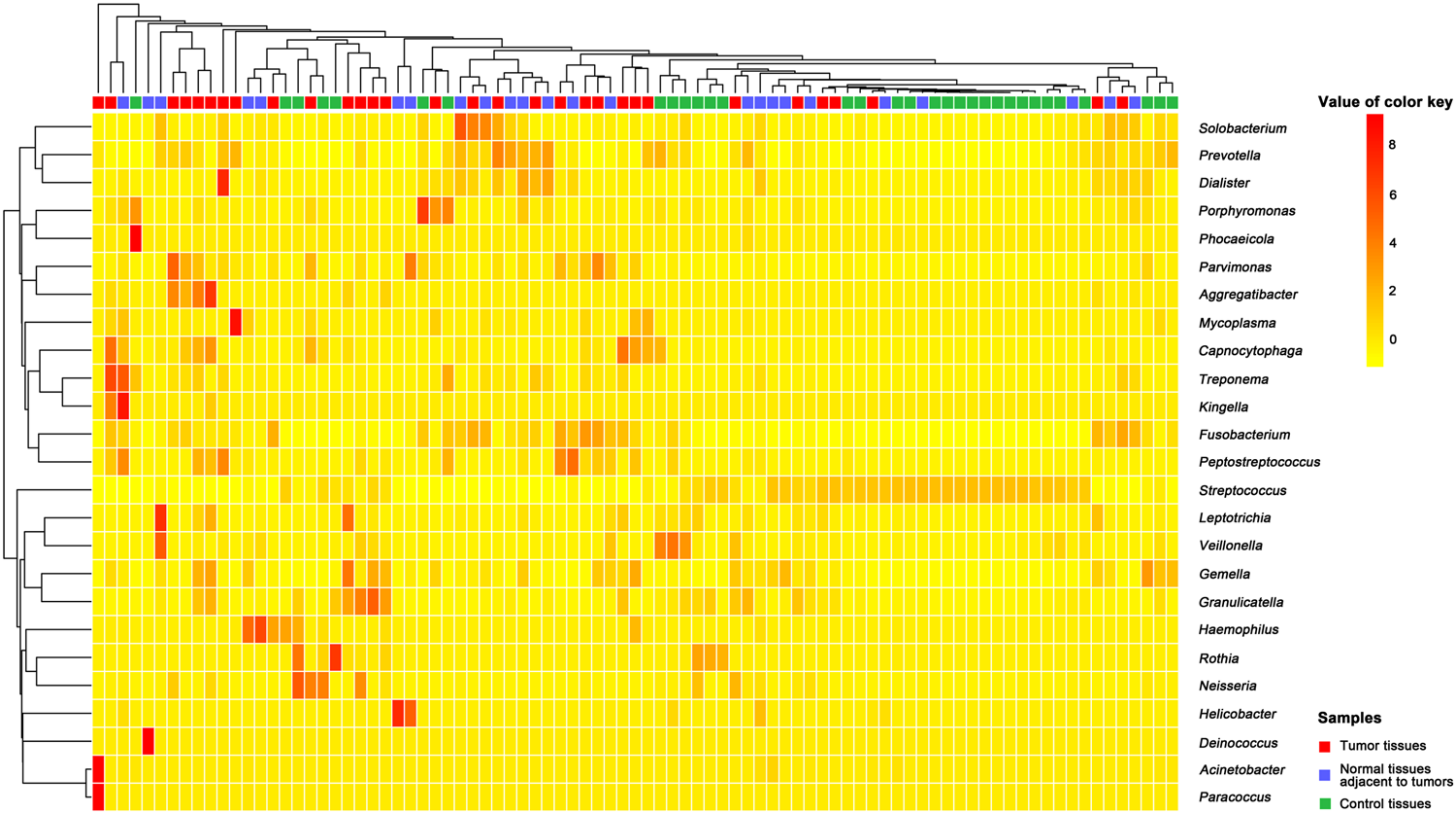
Hierarchical dendrogram showing taxonomic assignments from laryngeal samples. The heatmap summarizes the relative abundances of the 25 most abundant genera based on the analysis of laryngeal tissue samples. The legend in the upper-right corner of the figure shows the colors that correspond to the relative abundances of genera in each sample (expressed as a percentage of the total 16S rRNA sequences). The legend in the lower-right corner of the figure indicates the three groups of tissue samples. The tumor tissues and the normal tissues adjacent to tumor sites (NATs) were collected from laryngeal carcinoma patients, and the control tissues were collected from subjects with vocal cord polyps. The clusters of the three groups of samples based on bacterial communities are significant (*p* < 0.05), and the related *p* values were generated using a CrossMatch test.

We determined the microbial structures in the three groups of tissue samples and found significant differences in these niches. A heatmap colored according to the relative abundances was constructed to visualize the predominant genera in the three sample groups (the tumor tissues, the NATs, and the control tissues) (Fig. 2). We found that the control samples were separated from those of the tumor tissues and the NATs, and the genera *Streptococcus*, *Fusobacterium*, *Prevotella*, *Parvimonas*, *Peptostreptococcus*, *Dialister*, *Treponema*, *Capnocytophaga*, *Solobacterium*, and *Porphyromonas* contributed to this separation obtained through a cluster analysis (*p* < 0.05) (see Supplementary Table S3).

**Figure 3:**
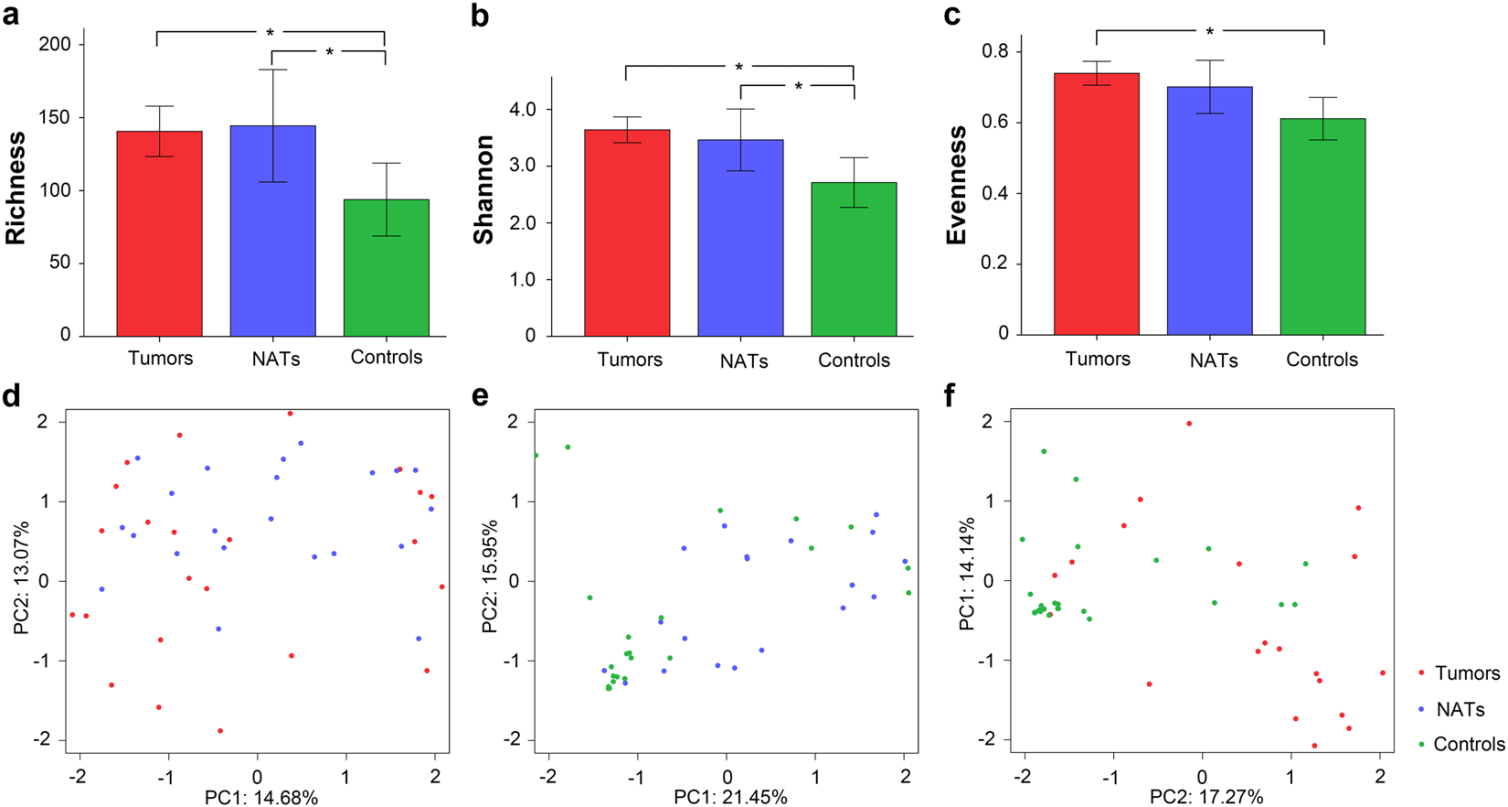
Comparative analyses of bacterial communities from the three groups of tissue samples from the larynx. The Richness (**a**), Shannon (**b**), and Evenness (**c**) of the samples from the tumor tissues, the normal tissues adjacent to the tumor sites (NATs) and control tissues are compared. The values present the means ± SEMs, * indicates *p* < 0.05. The tumor tissues and the NATs were collected from laryngeal carcinoma patients, and the control tissues were collected from control subjects. (**d**) A PCA based on weighted UniFrac distances was performed to analyze the community membership metrics of tumor tissues and NATs. (**e**) The bacterial community membership of NATs and control tissue samples was analyzed by PCA. (**f**) The bacterial community membership of the tumor tissues and the control tissue samples was analyzed by PCA. For the laryngeal carcinoma group, the communities from the tumor tissues appear in red, whereas communities from the NATs appear in blue. The communities from the control subjects appear in green. The statistical significance (*p* value) was analyzed using the R package CrossMatch based on the UniFrac sample distance.

The number of sequences per sample was normalized such that each sample contained 1000 sequences. We found higher bacterial Richness and Shannon diversity in the tumor samples and the NATs compared with the control samples (Richness: *p* = 0.002 and *p* = 0.021, respectively; Shannon diversity: *p* < 0.001 and *p* = 0.035, respectively) (Fig. 3a,b). Additionally, a higher bacterial Evenness was revealed in the bacterial communities in the tumor samples compared with that found in the control samples (*p* < 0.001) (Fig. 3c). A principal component analysis (PCA) revealed no separation of microbial genera between the tumor samples and the NATs (*p* = 0.78) (*p* value was analyzed by the R package CrossMatch) (Fig. 3d). Intriguingly, the microbial structures of these two sites differed from those of the control samples (the tumor tissues versus the control tissues, *p* < 0.001; the NATs versus the control tissues, *p* = 0.003) (*p* value was analyzed by the R package CrossMatch) (Fig. 3e,f). The 31 tumor samples and the 24 NATs were then combined to form the LSCC group (55 sample), and PCA were performed to compare the community membership metrics of these 55 LSCC samples and control samples (the statistical significance of p value was calculated using the R package CrossMatch). We found separation between the LSCC samples and the control samples based on the first two principal component (PC) scores, which accounted for 14.35% and 10.51% of the total variability at the genus level (*p* < 0.001) (see Supplementary Fig. S3a). We further evaluated the separation at the phylum level and found that the first two PC scores accounted for 29.43% and 15.67% of the total variability (*p* < 0.001) (see Supplementary Fig. S3b).

The relative abundances of several bacterial communities differed substantially among the three groups of tissue samples. An increase in the population size of the phylum *Fusobacteria* was found in the tumor samples and the NATs compared with the control samples, whereas decreases in the population sizes of the phyla *Firmicutes* and *Actinobacteria* were observed in these two groups of samples than those of control samples (*p* < 0.05) (Table 1). Moreover, the relative populations of the genera *Fusobacterium*, *Gemella*, *Capnocytophaga*, *Parvimonas*, *Aggregatibacter*, and *Peptostreptococcus* were significantly increased in the tumor samples compared with the control samples (*p* < 0.05). However, the relative abundance of the genus *Streptococcus* was decreased in the tumor tissues and the NATs compared with the control samples (*p* < 0.05). There was no difference between the tumor samples and the NATs (Table 1). Intriguingly, the relative abundance of the *Helicobacter* community was higher in the NATs (8.6%) compared with the tumor samples (0.1%) and the control samples (0.4%), although this difference was not significant (Table 1).

**Table 1:** Difference in the bacterial communities in the three types of laryngeal samples.

	Tumors^a^	NATs^b^	Controls^c^	*p1* ^d^	Adjusted *p1* ^e^	*p2* ^f^	Adjusted *p2* ^g^	*p3* ^h^	Adjusted *p3* ^i^
	%	%	%						
Phyla									
*Firmicutes*	35.9	36.9	63.6	0.898	0.898	0.001	0.002	0.003	0.012
*Fusobacteria*	25.4	18.4	7.6	0.246	0.328	0.001	0.002	0.021	0.042
*Actinobacteria*	1.5	0.7	4.7	0.132	0.328	0.085	0.085	0.034	0.045
Genera									
*Streptococcus*	25.3	31.3	65.4	0.468	0.572	<0.001	0.006	<0.001	0.011
*Fusobacterium*	26.7	17.9	6.9	0.193	0.275	0.001	0.006	0.023	0.081
*Gemella*	6.3	4.2	1.8	0.230	0.275	0.008	0.018	0.064	0.117
*Capnocytophaga*	2.4	0.5	0.3	0.014	0.147	0.007	0.018	0.525	0.577
*Parvimonas*	1.8	0.9	0.2	0.186	0.275	0.003	0.011	0.104	0.163
*Aggregatibacter*	1.4	0.09	0.04	0.040	0.147	0.032	0.050	0.221	0.270
*Peptostreptococcus*	0.7	0.6	0.1	0.660	0.726	0.019	0.035	0.164	0.226
*Helicobacter*	0.1	8.6	0.4	0.098	0.289	0.390	0.423	0.110	0.179

^a^The tumors were tumor tissue samples from laryngeal cancer patients. ^b^The NATs refered to normal tissues adjacent to the tumor sites from laryngeal cancer patients. ^c^The controls were control tissue samples from vocal cord polyps subjects. ^d^The comparison of bacterial communities between the tumor tissues and the NATs was performed, and the *p1* values were evaluated using Student’s t-test. ^e^The adjusted *p1* values were adjusted by the false discovery rate (FDR). ^f^The differences in the bacterial communities between the tumor tissues and the control tissues were assessed, and the *p2* values were evaluated using by Student’s t-test. ^g^The adjusted *p2* values were adjusted by the FDR. ^h^The differences in the bacterial communities between the NATs and the control tissues were analyzed, and the *p3* values were evaluated using Student’s t-test. ^i^The adjusted *p3* values were adjusted by the FDR.

The laryngeal communities in the LSCC patients presented different relative abundances compared with those of the control subjects. Because we found no difference in the community components and relative abundances between the 31 tumor samples and the 24 NATs, we removed the seven tumor samples that did not have related NATs and combined the remaining tumor tissues and their NATs to form another LSCC group (24 samples). The average relative abundances of the bacterial genera in each tumor tissues and its related NATs were obtained. The laryngeal microbial communities of the 24 LSCC samples were compared with those in the 32 control subjects, and we found that a broad array of bacterial populations presented significantly difference between these two groups. The relative abundance of the *Fusobacteria* phyla was enriched in the LSCC group, whereas *Firmicutes* was reduced in abundance (*p* < 0.05) (Table 2). Additionally, the genera *Fusobacterium*, *Prevotella*, *Parvimonas*, *Capnocytophaga*, *Dialister*, *Aggregatibacter*, *Peptostreptococcus*, *Solobacterium*, and *Selenomonas* were increased in the LSCC group compared with the control subjects, whereas *Streptococcus* was decreased in the LSCC group compared with the control subjects (*p* < 0.05). Interestingly, the relative abundance of *Helicobacter* was higher in the larynx of the LSCC group (4.3%) than in that of the control subjects (0.4%), but this difference was not significant (Table 2). A hierarchical clustering for assessing the structure of the bacterial genera in the 24 samples from LSCC patients and 32 control samples revealed that the control samples were separated from the samples from the LSCC patients. A dendrogram of the proportions of bacterial genera in the two groups was prepared to display the distance matrix (see Supplementary Fig. S4). The results revealed that the genera *Streptococcus*, *Fusobacterium*, *Prevotella*, *Parvimonas*, *Peptostreptococcus*, *Dialister*, *Treponema*, *Capnocytophaga*, *Solobacterium*, and *Porphyromonas* contributed to the separation of two groups (see Supplementary Table S4).

**Table 2:** Differences in the bacterial communities in the larynx of LSCC patients and control subjects.

	LSCC patients (%)^a^	Control subjects^b^	*p* ^c^	Adjusted *p* ^d^
Phyla				
*Fusobacteria*	24.7	7.6	<0.001	<0.001
*Firmicutes*	32	63.6	<0.001	0.001
Genera				
*Fusobacterium*	25.8	6.9	<0.001	<0.001
*Prevotella*	17.9	8.4	0.004	0.015
*Streptococcus*	24.5	65.4	<0.001	<0.001
*Parvimonas*	1.5	0.2	<0.001	0.002
*Capnocytophaga*	1.6	0.3	<0.001	0.02
*Dialister*	1.1	0.2	<0.001	0.024
*Aggregatibacter*	0.9	0.04	0.005	0.042
*Peptostreptococcus*	0.8	0.1	0.001	0.033
*Solobacterium*	0.5	0.2	0.001	0.046
*Selenomonas*	0.3	0.1	0.001	0.023
*Helicobacter*	4.3	0.4	0.077	0.129

^a^LSCC patients refered to laryngeal squamous cell carcinoma patients. ^b^The control subjects were subjects with vocal cord polyps. ^c^The *p* values were tested by Student’s t-test. ^d^The adjusted *p* values were adjusted by the false discovery rate (FDR).

The relative abundances of the bacterial communities in the LSCC patients and control subjects were analyzed again after adjusting for confounding factors. The differences in the bacterial communities between 24 laryngeal cancer patients and 32 control subjects with respect to each binary variable (sex, age, cigarette smoking, and alcohol drinking) were compared separately using metastats methods (see Supplementary Tables S5–S12). However, only two female LSCC patients and four female control subjects were recruited to this study. After adjusting for the variables of age, sex, smoking, and drinking, we observed using metastats methods that two bacterial phyla and two genera were different in the male population. Compared with that of the control subjects, the larynx of LSCC patients presented a reduced abundance of *Firmicutes* (30.8% vs 66.8%, *p* < 0.001) and an increased abundance of *Fusobacteria* (25.8% vs 5.3%, *p* < 0.001). The analysis of bacterial genera revealed that *Streptococcus* was reduced (23.0% vs 69.9%, p < 0.001) but *Fusobacterium* (27.1% vs 4.6%, *p* < 0.001) and *Prevotella* (17.4% vs 7.1%, *p* = 0.042) were increased in the larynx of LSCC patients compared with that of the control subjects.

## Discussion

In the study presented here, we examined the laryngeal microbiota patterns in 31 laryngeal cancer patients and 32 control subjects. The resulting microbiota structures suggested significant differences in the laryngeal communities in the tumor tissues, the NATs, and the control tissues. In addition to the observation of different laryngeal microbiota structural patterns being found in the LSCC patients compared with the control subjects, the relative abundances of certain communities largely differed in the LSCC patients relative to the control subjects. These observations, which constituted an incremental advance, verified our previous findings and indicated that an imbalance in the structure of laryngeal communities might be associated with laryngeal carcinoma.

To date, only two published articles conducted by researchers from other institutions examined the bacterial communities in the laryngeal tissue samples by analyzing the V3 - V5 regions of the 16S rRNA gene. Hanshew *et al*.^23^ examined the bacterial communities in the benign vocal cord lesions, and all tissue samples were collected from the vocal cords. Jetté *et al*.^28^ compared the bacterial communities of laryngeal tissue samples collected from the false vocal cords of volunteers based on reflux and smoking status. However, the structure of the bacterial communities in the throat has not been fully elucidated, and the associations between the microbiota structure and laryngeal carcinoma remain uncertain. In our pilot study, we analyzed the bacterial communities in the throat of patients with laryngeal carcinoma and vocal cord polyps by examining the 16S rRNA gene V1 - V2 regions, and these observations required further confirmation^26, 27^. In this study, we recollected laryngeal tissue samples and optimized the analysis methods, and we then fully characterized the microbiota structure by analyzing the 16S rRNA gene V3 region. The results of this study were more convincing and verified our previous observations that the microbiota in the throat of laryngeal carcinoma patients presented wide difference. These findings indicate that both the V1- V2 regions and the V3 region provide reliable information regarding taxonomic identity can be used for measurements of the richness and relative abundances in bacterial communities and for investigating the microbiota structure in the human throat.

The present study found that the community patterns presented differences among the three groups of laryngeal tissue samples. The structures of the bacterial communities in the tumor sites and the NATs of laryngeal carcinoma patients were different from those of the control samples collected from subjects with vocal cord polyp. The NATs used in the current study were one centimeter away from the corresponding tumor sites in the larynx, and evaluation of the laryngeal communities in the tumor sites and the NATs revealed no difference in α-diversity or β-diversity. The biological characteristics of the bacterial communities at the epithelial cells in these two niches of laryngeal cancer patients might be similar. However, Pushalkar *et al*.^15^ detected changes in the oral microbiota structures between the tumor and non-tumor tissues of OSCC patients and observed a shift in the oral bacterial communities that is likely correlated with OSCC. Even though the larynx and oral cavity are anatomical connected, the nature of microbial synergy in the human larynx niche might be different.

The detected alterations in the relative abundances of bacterial communities in the LSCC patients might be correlated with laryngeal carcinoma. Human bodies are composed of cells and microbes that exist in intrinsically complicated structures, with microbes effectively constituting another human organ^1^. The structure of this organ arises from the dynamics of community selection and competition^1, 29^. The microbiota composition might define the relative prevalences of specific laryngeal microbes, and alterations in these bacterial communities, which involve increases or decreases in the abundance of certain bacteria in their niche sites, likely enhances the susceptibility of the throat to disease. *Fusobacterium* possesses adhesive and invasive activities due to the membrane occupation and recognition nexus (MORN2), fadA, and radD genes, which encode adhesive and invasive proteins^30^. These proteins attach to the cell surface and bind to E-cadherin^18^. These proteins then invade the cells through the internalization of E-cadherin and clathrin and activate the β-catenin phosphorylation signaling pathway^18, 31^. Finally, *Fusobacterium* induces tumorigenesis by stimulating the expression of IL-18, PPARγ, and CCR2, activating the pro-oncogene BRAF, and inducing mutations in KRAS, P65, P53, CHD7/8, and mutL homolog 1 (MLH1), thereby leading to microsatellite instability (MSI)^30–33^. *Prevotella* predominates in the oral cavity and the upper respiratory tract, and contributes to chronic inflammatory infections. This genus also plays a role in glucose metabolism and is anticorrelated with other bacterial communities in term of relative abundance^34^. Intriguingly, the *Helicobacter* community is also distributed in the laryngeal mucosal niche. Although the differences did not reach statistical significance, *Helicobacter* was found more frequently in the laryngeal carcinoma patients than in the control subjects, a result that is similar to previous findings^35^. Alterations in certain bacteria in complex communities are also partially driven by *Helicobacter pylori (H. pylori)* ^36^. One hypothesis is that *H. pylori* recruits T lymphocytes and induces the release of cytokines and chemokines^36^, and to further promote inflammatory responses, *H. pylori* disrupts the epithelial barrier function and influences the overgrowth or disappearance of specific communities^36^. Moreover, *H. pylori* can reduce the expression of mutS homolog 2 and MLH1 and can initiate laryngeal carcinoma^37^. According to our results, the main bacterial community in the laryngeal mucosa niche is *Streptococcus*. This populations establish biofilms on the surface of the laryngeal mucosa through multifactorial cooperation and mechanisms that include the LuxS and AI-2 signaling pathways, the sensing system involving CbpA binding to the laminin receptor and pIgR, and the homodimerization of the BR domain of PsrP^38^. Microbiota with particular physiological or pathological functions can broadly influence the biological behavior of specific bacteria through complicated species-microbiota interactions^39^. *Streptococcus* can additionally interact with other microbial communities to achieve functions such as generating the products of saccharolytic short-chain organic acids from carbohydrates, decreasing the pH of the local environment, repressing the phosphoenolpyruvate-dependent phosphotransferase system, and increasing oxidative stress protection^40, 41^. A recent study also found that *Streptococcus* can produce and release an anticancer factor (tenovin-6-like molecule) to induce the growth inhibition and coccoid conversion of *H. pylori* ^42^. Together, these observations indicate that the bacterial species residing on laryngeal mucosal surfaces maintain constant contact with each other and host cells, yielding a mutual biological interaction between the microbiota and the host. Even though this study was a cross-sectional study, the results suggest that the loss of balance in the microbiota achieved due to weakening or strengthening of different competing subpopulations might serve as a causative factor for laryngeal carcinoma. However, this suggestion has to be confirmed by further studies.

In this study, only two women with laryngeal cancer were recruited. Laryngeal carcinoma is predominantly a cancer of men and is rare in women^20, 43^. We adjusted other risk factors to avoid potential bias and found that the relative abundances of *Streptococcus* and *Fusobacterium* were reduced and enriched, respectively, in the larynx of male laryngeal cancer patients. Further studies with greater numbers of patients are required to avoid potential bias.

In summary, we compared the microbiota structures in the larynx between laryngeal cancer patients and control subjects and identified differences in the microbial community structures in the larynx that likely contributed to the development of laryngeal carcinoma. These findings provide a foundation for future studies of the microbe-microbiota-host interplay and for the investigation of their exact roles in the initiation and development of laryngeal carcinoma.

## Methods

### Study area and subject recruitment

The study was performed with samples collected from 63 patients who underwent surgery in the Department of Otolaryngology at the Eye, Ear, Nose, and Throat Hospital of Fudan University. The study subjects were enrolled between January 2011 and March 2012 from two groups of patients, including 31 LSCC patients and 32 subjects with vocal cord polyps. Six females and 57 males were recruited, and the subjects ranged in age from 35 to 75 years (mean 56.6 ± 4.32 years). The cigarette smoking and alcohol consumption of each subject were also recorded. Tumor stage was determined according to the International Union Against Cancer TNM classification system, 6th Edition^44^. Subjects who had taken any antibiotics or antimycotics and/or hormone compounds in the past 30 days were excluded. The LSCC patients underwent total laryngectomy surgery, and the subjects with vocal cord polyps were treated with direct laryngoscope surgery. The Ethics Committee at the Eye, Ear, Nose, and Throat Hospital of Fudan University approved the protocols and consent forms of this study. The methods were performed in accordance with the approved guidelines and regulations. All of the subjects provided informed consent to participate in this study.

### Tissue sample collection

We sampled the tumor tissues and the NATs from the LSCC patients and collected control tissues from the subjects with vocal cord polyps. The diagnosis of each patient was confirmed by histopathology. To avoid contamination, we obtained all of the tissue samples immediately after surgery in a laminar-flow operating room. The tumor tissues were sampled from the superficial layer of each tumor site, and the NATs were collected from areas located at least one centimeter away from the tumor site^45^. The control tissues were obtained from superficial layer of the polyps of the patients with vocal cord polyps, which is the only way to collect non-tumor tissues from the larynx of subjects without tumors, as noted in previous studies^23, 46^. Although vocal cord polyps are not healthy tissues, the results of previous studies indicated that these vocal cord polyps can constitute meaningful control samples^26, 27, 47^. In total, 31 tumor tissues and 24 NATs were obtained. Matched NATs were not acquired for seven tumor tissues because the negative margins were less than one centimeter away from each tumor site. All samples were preserved in microcentrifuge tubes (Axygen, Shanghai) at −80 °C for subsequent DNA extraction.

### DNA extraction and amplification

Whole genomic DNA was extracted from the tissues using a QIAGEN DNeasy kit (QIAGEN, Germany), and the manufacturer’s protocol was modified to allow the recovery of bacterial DNA. Briefly, a lytic enzyme cocktail mix (Sigma-Aldrich, USA) and 0.1-mm-diameter stainless zirconia/silica beads (BioSpec Products, USA) were used to maximize the DNA product yield^48^. After a tube of tissues was thawed on ice for five min, 200 μl of ATL buffer was added to the tube before vortexing for 15 s. After 20 μl of Proteinase K was added to the tube, the tube was vortexed thoroughly and incubated at 56 °C overnight, and 59 μl of the enzyme cocktail and 41 μl of TE buffer (10 mmol/l Tris-HCl + 50 mmol/l EDTA, pH 8.0) were added to the sample. Beads were then added to the sample for bead beating, and 500 μl of AL buffer was then added. The sample was then vortexed for 15 s and incubated for 10 min at 70 °C. The supernatant was transferred to a new 1.5-ml microcentrifuge tube, and the beads were kept in the original tube. After 500 μl of 100% ethanol was added to the tube, the tube was vortexed for 15 s, and 500 μl of the AW1 buffers and 500 μl of the AW2 buffers were added to purify the genomic DNA according to the manufacturer’s instructions. Finally, the AE buffer was used to elute the DNA^48^, and the purified DNA was stored at −80 °C prior to further analysis.

The V3 region of the bacterial 16S rRNA gene was amplified from the tissue samples by the polymerase chain reaction (PCR) using two sets of universal bacterial primers (Sangon, Shanghai)^26, 49^. The PCR program was as follows: 95 °C for 2 min followed by 30 cycles of 95 °C for 20 sec, 56 °C for 30 sec, and 72 °C for 5 min. The Ex Taq PCR mixture (Takara, Dalian) was used to amplify the 16S rRNA genes. The PCR products were then purified and sent for further sequence analysis.

The appropriate negative controls were used for the genomic DNA extraction, purification, and PCR amplification.

### Sequencing application and data analyses

The amplicons of the 16S rRNA genes from different samples were mixed at equal ratios and pyrosequenced using a GS FLX platform. Single-stranded template DNA (sstDNA) libraries were generated from the prepared DNA using a GS DNA Library Preparation Kit (Roche Applied Science). The sstDNA libraries were clonally amplified in a bead-immobilized form using the GS emPCR Kit (Roche Applied Science) and sequenced using the 454 Genome Sequencer FLX Titanium platform. Negative controls were used for preparation of the 16S rRNA gene library, and the pyrosequencing was performed at the Chinese National Human Genome Sequencing Center (Shanghai, China).

Sequences that did not meet the minimum standard of quality were discarded using protocols described previously^26, 48^. A denoising strategy was applied using mothur based on Chris Quince’s PyroNoise algorithm^50^. UCLUST software was used to identify OTUs at a level of 97% similarity. The Richness was analyzed based on the number of observed OTUs, the Evenness was measured as *E*_*Shannon*_ = *D*_*Shannon*_/ln(*S*), and the Shannon diversity was evaluated using the non-parametric Shannon Index^6^. The Shannon diversity, Simpson diversity, Evenness, ACE, Chao1, and Good’s coverage indices were calculated using mothur^51^ (v.1.27.0, http://www.mothur.org/wiki/Main_Page). The online software RDP Classifier was used to assign the sequences to phylogenetic taxonomies based on the Ribosomal Database Project^52, 53^; the sequences were assigned to hierarchical taxa under a bootstrap cut-off condition of 80%. A heatmap showing the distance of the sample clusters was generated using the Heatmap.2 software package, and the most abundant sequences at the genus level were analyzed with UniFrac software^54^. The significant differences in the microbial community compositions among the different sample categories were determined using the metastats methods (http://metastats.cbcb.umd.edu/detection.html), and Student’s t-test was adjusted using the false discovery rate (FDR). The complete linkage algorithm was used for clustering, which was analyzed by PCA using the R program. The relative abundances of the bacterial genera in the tissue samples from LSCC patients and control subjects were analyzed by weighted hierarchical cluster analysis using the average linkage method. The distances between the samples were calculated using Squared Euclidean distances and was displayed graphically as a dendrogram.

### Data availability

The dataset(s) of the V3 region supporting the results of this article are available in the NCBI Sequence Read Archive (SRA) under accession number SRP059583.

## Electronic supplementary material

Supplementary information
