# Supplementary material for: Alterations of microbiota structure in the larynx relevant to laryngeal carcinoma
Source: Sci Rep. 2017 Jul 14;7:5507. doi: 10.1038/s41598-017-05576-7 (PMC5511217; doi:10.1038/s41598-017-05576-7)
Supplement: Supplementary file 1 — Supplementary information [file 41598_2017_5576_MOESM1_ESM.pdf]

Supplementary Information

Title: Alterations of microbiota structure in the larynx relevant to laryngeal carcinoma

Authors: Hongli Gong, Yi Shi, Xiyan Xiao, Pengyu Cao, Chunping Wu, Lei Tao, Dongsheng Hou, Yuezhu Wang, Liang Zhou

Supplementary tables

	LSCC subjects	Control subjects	*p*
Gender			
male	29	28	0.672[a]
female	2	4	
Age			
≤ 60 years	11	14	0.503[b]
> 60 years	20	18	
Smoking			
never smoking	9	17	0.052[b]
smoking	22	15	
Drinking			
never drinking	13	20	0.102[b]
drinking	18	12	
Tumor type			
supraglottic	11		
glottic	20		
T stages			
T1 and T2	12		
T3 and T4	19		

Table S1. Clinical characteristics of recruited patients in this study. [a] *p* value was tested from Fisher's Exact test. [b] *p* values were tested from Pearson Chi-Square test

OTUs	Chao1	95% C.I.		ACE	95% C.I.		Simpson	95% C.I.		Shannon	95% C.I.		Coverage	Evenness
8887	13789.29	13359.53	14260.34	15809.74	15482.02	16153.74	0.0043	0.00424	0.00436	6.874	6.866	6.883	0.98	0.76

Table S2. Evaluating indices of the 16S rRNA gene libraries at a level of 97% similarity from the pyrosequencing analysis in this study

	Tumor samples	NATs[a]	Control samples	F[b]	*p*
Genera					
Streptococcus	25.3	31.3	65.4	249.9	<0.001
Fusobacterium	26.7	17.9	6.9	27.0	<0.001
Prevotella	15.5	16.8	8.4	14.8	<0.001
Parvimonas	1.8	0.9	0.2	8.8	<0.001
Peptostreptococcus	0.7	0.6	0.1	6.0	0.004
Dialister	1	1	0.2	4.9	0.01
Treponema	0.8	0.7	0.2	4.7	0.012
Capnocytophaga	2.4	0.5	0.3	4.6	0.012
Solobacterium	0.3	0.7	0.2	4.6	0.012
Porphyromonas	1.4	1.3	2.6	3.6	0.031

Table S3. **The main bacterial genera contributing to separation among the tumor tissue, the NATs, and the control tissues.** [a] NATs were the normal tissue adjacent tumor sites. [b] F tests were used for descriptive purpose of different genera that contributed to three groups of cluster analyses, and these results were analyzed using means clustering analysis

	LSCC patients (%)[a]	control subjects (%)	F[b]	*p*
Genera				
Streptococcus	25.5	65.4	237.4	<0.001
Fusobacterium	25.8	6.9	51.9	<0.001
Prevotella	17.9	8.4	27.0	<0.001
Parvimonas	1.5	0.2	17.3	<0.001
Dialister	1.1	0.2	10.6	0.002
Mogibacterium	0.1	0.1	10.1	0.003
Solobacterium	0.5	0.2	6.7	0.013
Treponema	0.9	0.2	6.4	0.014
Tannerella	0.03	0.004	6.1	0.016
Peptostreptococcus	0.8	0.1	6.0	0.018
Porphyromonas	1	2.6	5.3	0.026
Mycoplasma	1.4	0.1	4.5	0.038

Table S4. The main bacterial genera contributing to separation between LSCC patients and control subjects. [a] LSCC were patients with laryngeal squamous cell. [b] F tests were used for descriptive purpose of different genera that contributed to two groups of cluster analyses, and these results were analyzed using means clustering analysis

	LSCC patients (%)[a]	Control subjects (%)	p^{b}
Phyla			
Firmicutes	31.0	67.2	0.012
Fusobacteria	27.8	7.1	0.001
Bacteroidetes	21.2	10.1	0.029
Spirochaetes	0.4	0.03	<0.001
Genera			
Fusobacterium	27.9	6.1	0.001
Prevotella	16.9	5.7	0.028
Streptococcus	23.5	70.6	0.003
Gemella	3.4	1.9	0.013
Parvimonas	1.8	0.4	0.002
Capnocytophaga	1.2	0.1	0.019
Peptostreptococcus	0.8	0.04	0.004
Dialister	0.8	0.2	0.006
Solobacterium	0.6	0.2	0.042
Treponema	0.4	0.04	0.001

Table S5. Differences in the bacterial communities between LSCC patients and control subjects whose ages were less than 60 years. [a] LSCC were patients with laryngeal squamous cell carcinoma. [b] *p* values were calculated using metastats methods

	LSCC patients (%)[a]	control subjects (%)	*p*[b]
Phyla			
Fusobacteria	22.0	7.9	0.002
Firmicutes	35.2	60.9	0.018
Bacteroidetes	24.9	14.3	0.022
Spirochaetes	0.9	0.3	<0.001
Tenericutes	0.9	0.1	0.024
Genera			
Fusobacterium	23.8	7.5	0.001
Prevotella	19.2	8.5	0.036
Streptococcus	28.0	61.4	0.008
Gemella	4.9	1.7	0.001
Capnocytophaga	1.7	0.5	<0.001
Dialister	1.3	0.1	0.002
Treponema	1.2	0.4	<0.001
Mycoplasma	1.2	0.1	0.024
Parvimonas	1.2	0.1	<0.001
Aggregatibacter	1.1	0.05	0.01
Solobacterium	0.5	0.1	0.004

Table S6. Differences in the bacterial communities between LSCC patients and control subjects whose ages were more than 60 years. [a] LSCC were patients with laryngeal squamous cell carcinoma. [b] *p* values were calculated using metastats methods

	LSCC patients (%)[a]	control subjects (%)	p [b]
Phyla			
Fusobacteria	31.5	9.8	0.001
Firmicutes	22.2	59.2	0.005
Actinobacteria	0.5	3.8	0.038
Spirochaetes	0.5	0.3	<0.001
Genera			
Fusobacterium	34.0	9.1	<0.001
Prevotella	18.8	8.1	0.029
Streptococcus	14.7	60.3	0.003
Gemella	2.8	1.8	0.01
Parvimonas	2.4	0.3	<0.001
Capnocytophaga	1.0	0.3	0.011
Dialister	1.0	0.2	0.006
Peptostreptococcus	0.7	0.2	0.031
Aggregatibacter	0.7	0.04	0.008
Treponema	0.6	0.3	0.001
Solobacterium	0.4	0.1	0.004
Staphylococcus	0.3	0.01	0.006

Table S7. Differences in the bacterial communities between LSCC patients and control subjects who did not drinking alcohol. [a] LSCC were patients with laryngeal squamous cell carcinoma. [b] *p* values were calculated using metastats methods

	LSCC patients (%)[a]	control subjects	p^b
Phyla			
Fusobacteria	17.8	3.8	0.007
Firmicutes	41.0	71.1	0.04
Bacteroidetes	23.5	7.4	0.006
Spirochaetes	0.9	0.03	0.004
Genera			
Fusobacterium	17.7	3.2	0.004
Prevotella	19.0	7.9	0.035
Streptococcus	33.6	74.0	0.006
Gemella	5.7	1.5	0.002
Capnocytophaga	1.9	0.3	0.014
Treponema	1.1	0.04	0.003
Parvimonas	0.7	0.05	0.003
Campylobacter	0.2	0.02	0.043

Table S8. Differences in the bacterial communities between LSCC patients and control subjects who drank alcohol. [a] LSCC were patients with laryngeal squamous cell carcinoma. [b] *p* values were calculated using metastats methods

	LSCC patients (%)[a]	Control subjects	p^b
Phyla			
Fusobacteria	27.0	9.4	0.008
Firmicutes	24.0	58.7	0.007
Genera			
Fusobacterium	28.9	8.7	0.006
Prevotella	25.7	9.6	0.02
Streptococcus	17.5	60.3	0.045
Parvimonas	1.5	0.4	0.002
Capnocytophaga	1.4	0.4	0.031
Dialister	1.2	0.3	0.005
Solobacterium	0.5	0.1	0.016

Table S9. Differences in the bacterial communities between LSCC patients and control subjects who did not smoke. [a] LSCC were patients with laryngeal squamous cell carcinoma. [b] *p* values were calculated using metastats methods

	LSCC patients (%)[a]	control subjects	pb
Phyla			
Fusobacteria	23.1	5.5	0.001
Firmicutes	37.7	69.3	0.003
Bacteroidetes	17.4	8.4	0.01
Spirochaetes	0.9	0.02	<0.001
Genera			
Fusobacterium	23.3	4.8	<0.001
Prevotella	14.3	7.2	0.042
Streptococcus	28.6	71.2	<0.001
Gemella	5.9	1.3	0.001
Capnocytophaga	1.7	0.2	0.002
Parvimonas	1.5	0.06	<0.001
Porphyromonas	1.1	0.4	0.016
Aggregatibacter	1.3	0.03	0.001
Treponema	1.1	0.03	<0.001
Peptostreptococcus	0.9	0.09	0.002
Solobacterium	0.6	0.2	0.032
Selenomonas	0.3	0.01	0.009
Campylobacter	0.2	0.04	0.013

Table S10. Differences in the bacterial communities between LSCC patients and control subjects who smoked. [a] LSCC were patients with laryngeal squamous cell carcinoma. [b] *p* values were calculated using metastats methods

	LSCC patients (%)[a]	control subjects	p[b]
Phyla			
Fusobacteria	25.8	5.3	<0.001
Firmicutes	30.8	66.8	<0.001
Bacteroidetes	22.7	11.5	0.001
Spirochaetes	0.7	0.1	<0.001
Cyanobacteria	0.4	0.03	0.049
Genera			
Fusobacterium	27.1	4.6	<0.001
Prevotella	17.4	7.1	0.002
Streptococcus	23.0	69.9	<0.001
Gemella	4.5	1.6	<0.001
Parvimonas	1.6	0.1	<0.001
Mycoplasma	1.3	0.01	0.007
Dialister	1.2	0.08	<0.001
Treponema	0.9	0.1	<0.001
Peptostreptococcus	0.8	0.07	<0.001
Solobacterium	0.6	0.1	0.001
Aggregatibacter	0.6	0.03	0.006
Streptophyta	0.4	0.02	0.016
Selenomonas	0.3	0.05	0.001
Campylobacter	0.2	0.4	0.025
Serratia	0.2	0.03	0.031
Staphylococcus	0.2	0.01	0.005

Table S11. Differences in the bacterial communities between LSCC patients and control subjects who were males. [a] LSCC were patients with laryngeal squamous cell carcinoma. [b] p values were calculated using metastats methods

	LSCC patients (%)[a]	Control subjects (%)	p^b
Genera			
Capnocytophaga	3.9	0.01	0.049
Haemophilus	0.7	0.04	0.049

Table S12. Differences in the bacterial communities between LSCC patients and control subjects who were females. [a] LSCC were patients with laryngeal squamous cell carcinoma. [b] *p* values were calculated using metastats methods

Supplementary figures

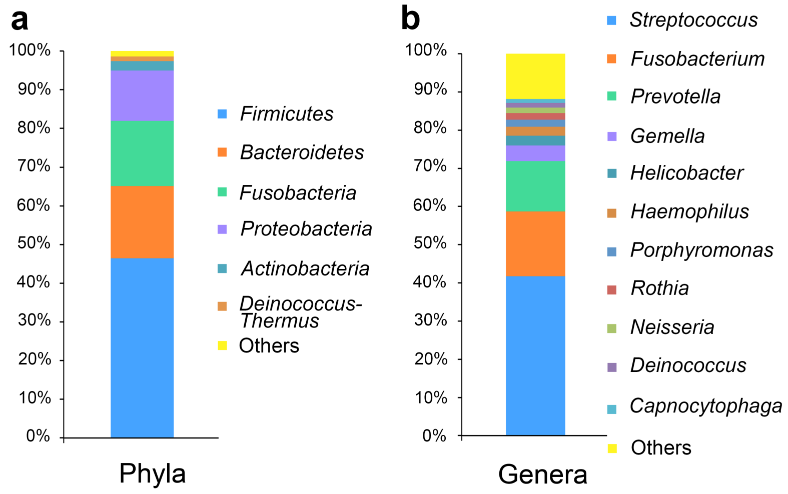

Figure S1. Abundances of dominant bacterial communities in the larynx. (a) Abundances (% of total 16S rRNA sequences) of the predominant bacterial phyla in the laryngeal mucosa of 63 subjects (including 31 tumor samples, 24 normal tissues adjacent to the tumor sites, and 32 control samples). **(b)** Main bacterial genera in the laryngeal mucosa of 63 subjects.

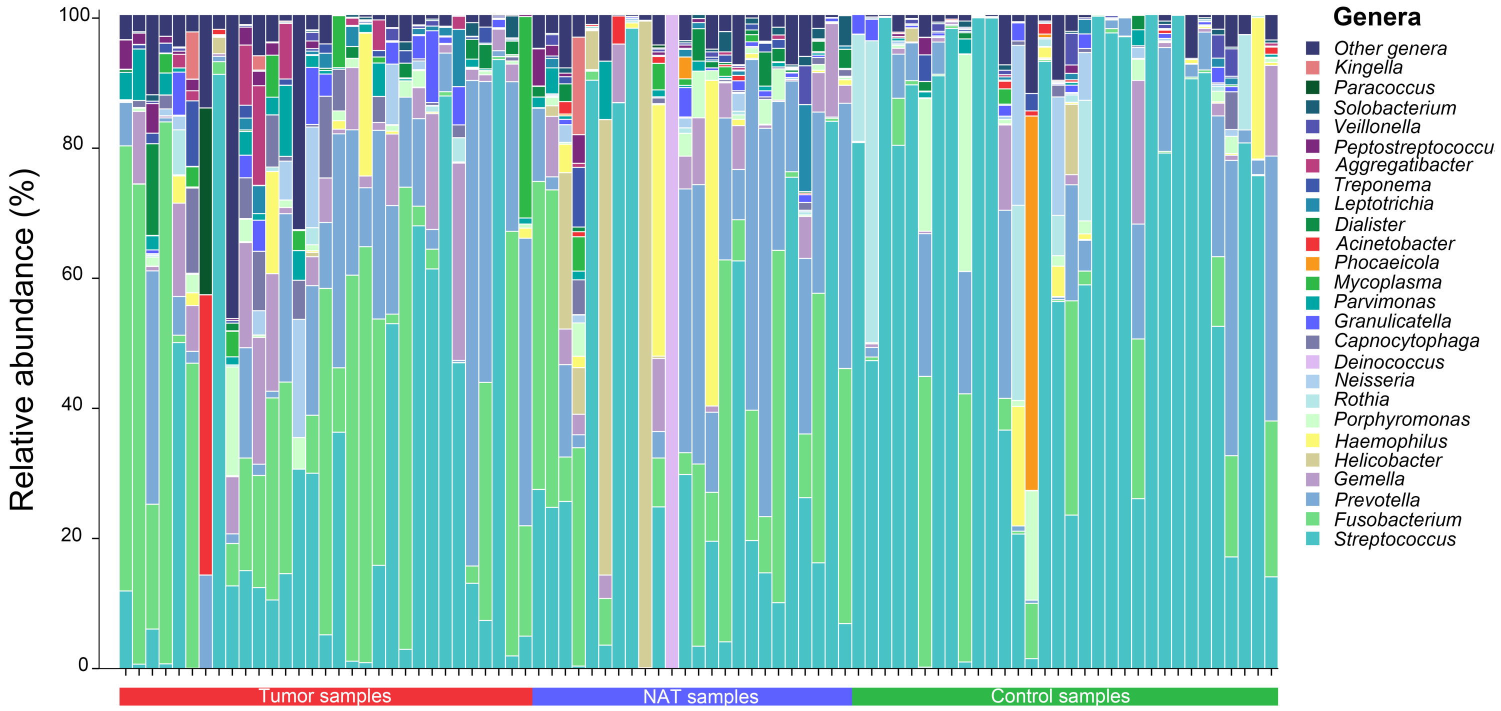

Figure S2. Distributions of the predominant bacterial genera at the laryngeal sites. The stacked columns for each of the tissue samples indicate the relative abundance distributions of the 25 main genera from three groups of samples. The red bar at the bottom of the legend indicates the tumor tissue samples. The blue bar indicates the normal tissues adjacent to the tumor sites (NATs) samples. The green bar indicates vocal cord polyps samples. The bars on the right of the legend indicate the main bacterial genera.

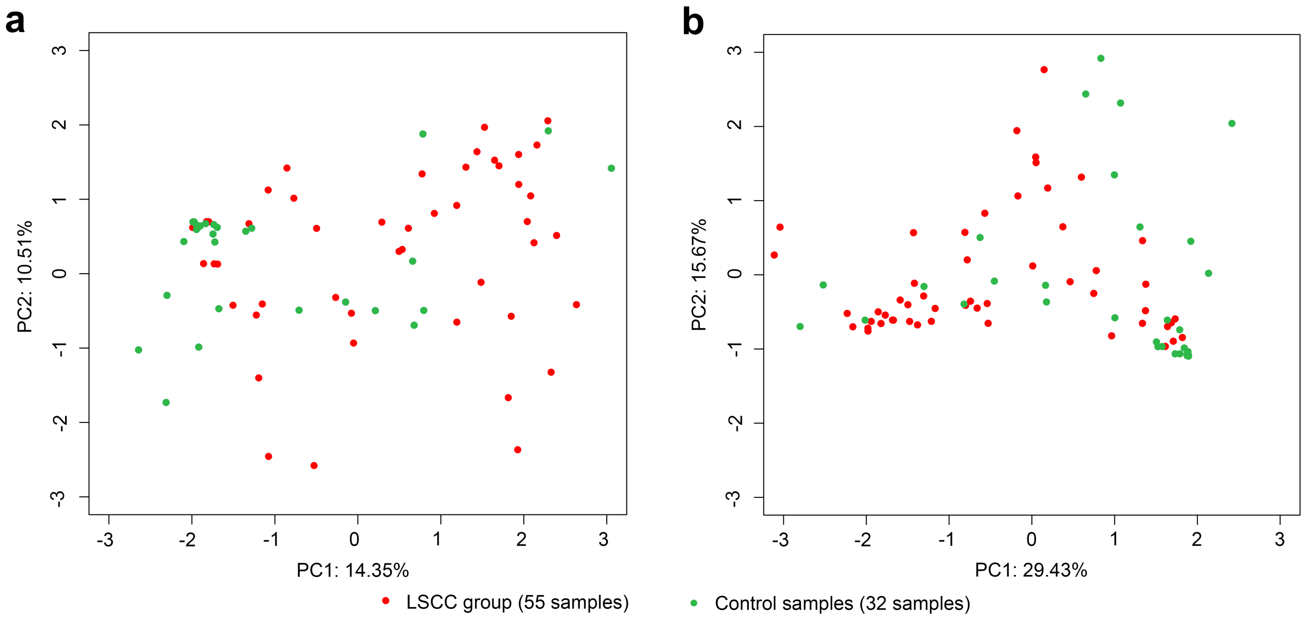

Figure S3. Comparative analyses of the bacterial communities in LSCC samples and the control samples. Principal component analysis (PCA) based on the weighted UniFrac distance were used to analyze the bacterial genera **(a)** and bacterial phyla **(b)** membership metrics of the LSCC group (including 31 tumor samples and 24 normal tissues adjacent to the tumor sites (NATs)) and control samples (32 control samples). The red point at the bottom of the figure indicated the LSCC patients, and the green point indicated the control subjects. The statistical significance (*p* value) was analyzed using the R package CrossMatch based on the UniFrac sample distance. LSCC, laryngeal squamous cell carcinoma.

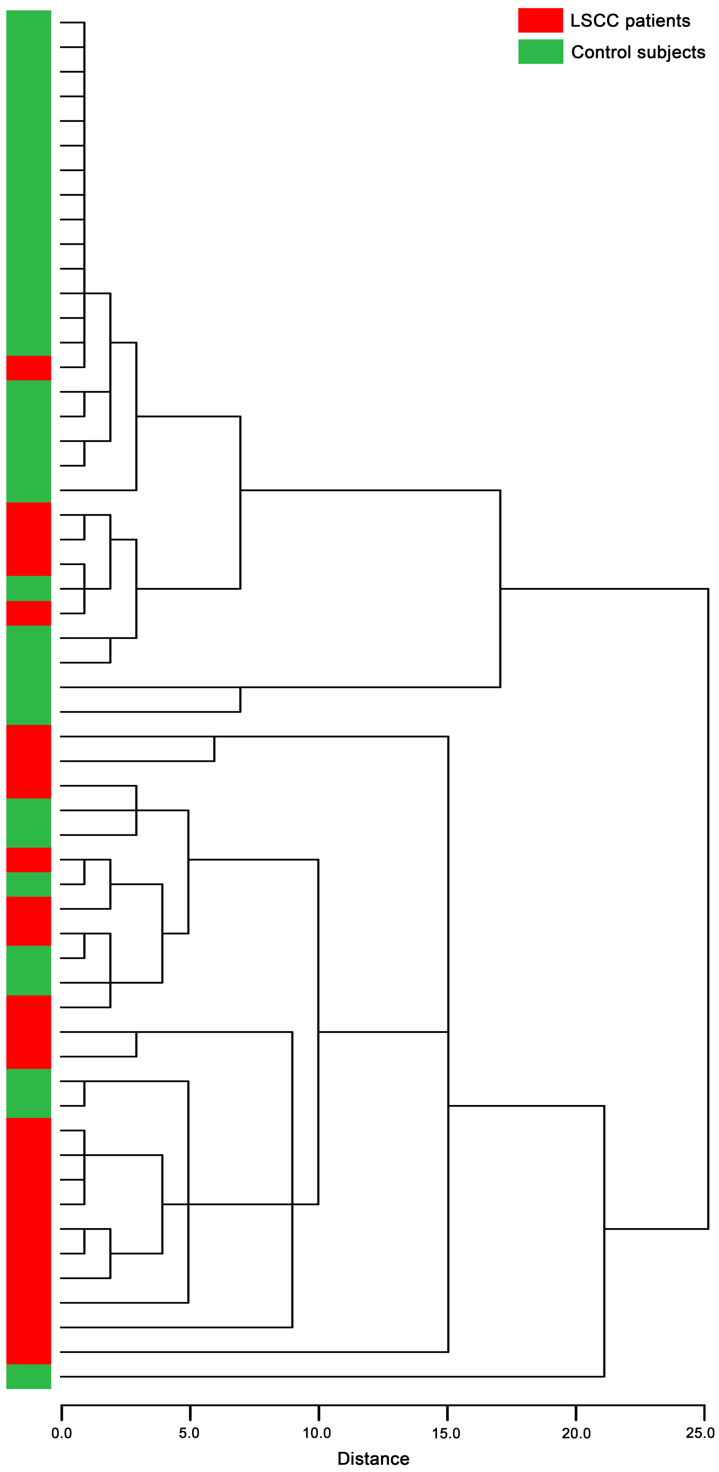

Figure S4. Hierarchical clustering of two groups of sample profiles. Visualization of similarities between LSCC patients (24 LSCC samples) and control subjects (32 control samples) by hierarchical clustering analysis with the average linkage method and based on the Squared Euclidean distances. Samples from the control subjects were separated from those of LSCC patients. The scale bar indicated a weighted distance from 0 to 25. The red bar at the top-right of the figure indicated the LSCC patients, and the green bar indicated the control subjects.
